# Assessing the Effects of Alloxydim Phototransformation Products by QSAR Models and a Phytotoxicity Study

**DOI:** 10.3390/molecules23050993

**Published:** 2018-04-24

**Authors:** Juan J. Villaverde, Inés Santín-Montanyá, Beatriz Sevilla-Morán, José L. Alonso-Prados, Pilar Sandín-España

**Affiliations:** 1 Unit Plant Protection Products, DTEVPF, INIA. Crta. La Coruña, Km.7.5, 28040 Madrid, Spain; juanjose.villaverde@inia.es (J.J.V.); bsmoran@inia.es (B.S.-M.); prados@inia.es (J.L.A.-P.); 2 Department of Plant Protection, INIA. Crta. La Coruña Km. 7.5, 28040 Madrid, Spain; isantin@inia.es

**Keywords:** degradation, herbicide, transformation products, phytotoxicity, quantitative structure–activity relationship

## Abstract

Once applied, an herbicide first makes contact with leaves and soil. It is known that photolysis can be one of the most important processes of dissipation of herbicides in the field. However, degradation does not guarantee detoxification and can give rise to byproducts that could be more toxic and/or persistent than the active substance. In this work, the photodegradation of alloxydim herbicide in soil and leaf cuticle surrogates was studied and a detailed study on the phytotoxicity of the main byproduct on sugar beet, tomato, and rotational crops was performed. Quantitative structure–activity relationship (QSAR) models were used to obtain a first approximation of the possible ecotoxicological and environmental implications of the alloxydim and its degradation product. The results show that alloxydim is rapidly degraded on carnauba and sandy loam soil surfaces, two difficult matrices to analyze and not previously studied with alloxydim. Two transformation products that formed in both matrices were identified: alloxydim Z-isomer and imine derivative (mixture of two tautomers). The phytotoxicity of alloxydim and the major byproduct shows that tomato possesses high sensitivity to the imine byproduct, while wheat crops are inhibited by the parent compound. This paper demonstrates the need to further investigate the behavior of herbicide degradation products on target and nontarget species to determine the adequate use of herbicidal products to maximize productivity in the context of sustainable agriculture.

## 1. Introduction

Public concern about the use of synthetic chemicals on crops is growing, and the legislative requirements from authorities for the registration of agrochemicals are becoming stricter [1,2]. The use of pesticides is so intense around the world that pesticide residues can usually be found in environmental compartments [2,3,4]. In this sense, the general trend of companies is to develop pesticides with low application doses and short-lived time to diminish their persistence in the environment. However, in some cases, this advantageous feature can present drawbacks including the generation of transformation products and metabolites by degradation processes, which can be more toxic and/or mobile than their parent pesticides [5,6,7,8]. Also, although pesticides are intended to protect crops, they or their degradation products may actually affect nontreated neighboring crops, rotational crops, or successive crops (nontarget species), resulting in alterations of the equilibrium of agro systems in the mid to long term [9,10].

In this scenario, the correct use of pesticides, together with a correct active substance selection taking into account its environmental behavior and effects on human and animal health and the environment, is of great importance. Alloxydim is a cyclohexanedione-oxime herbicide that is used for monocotyledonous weed control and cereal sprouts in broadleaf crops, such as sugar beet, soya bean, and pea [11]. It is a post-emergence herbicide applied by foliar spray. Degradation studies of herbicides such as alloxydim on leaves and soil surfaces are poorly documented [12,13,14,15,16]. The heterogeneity of the surfaces and the complex light phenomena make experiments quite difficult. Previous studies by our group showed that alloxydim rapidly photodegrades in water [17,18,19]. However, before entering into the aqueous compartments, the herbicide, after spraying, comes in contact with soil and plant leaf surfaces and is subjected to sunlight irradiation. The consequences could be a reduction of efficacy and the appearance of degradation products of unknown physicochemical, biological, and environmental properties that can be absorbed by the plant through the leaves or the roots in soil. Therefore, it is important to design experiments to study the behavior of pesticides under scenarios that mimic these environmental matrices, as an important step to provide a better knowledge of pesticides in the field, to improve their impacts and risk assessments.

Computational studies based on quantitative structure–activity relationship (QSAR) models can be used to predict key properties of pesticides and their transformation products and hence the eco/toxicological and environmental fate potential of these compounds. The quantitative associations provided by QSAR models are of special interest in the study of transformation products, as many of them are not available commercially and are difficult to isolate [20,21].

In this context, the photodegradation of alloxydim in carnauba wax films and sandy loam soil surfaces as surrogates of leaf cuticle and real soils was investigated. The photoproducts formed were identified, and their evolution in the different matrices was studied. A detailed study to determine the phytotoxicity of the main byproduct on sugar beet, tomato, and rotational crops was performed by the use of plant growth bioassays, which are widely accepted to monitor growth responses to different chemicals [22,23,24]. Finally, in silico QSAR models were used to estimate further physicochemical, environmental, and ecotoxicological endpoints of alloxydim and its main byproducts in order to perform an initial risk assessment.

## 2. Results and Discussion

### 2.1. Photolytic Behavior of Alloxydim and Byproduct Identification

Prior to photolysis experiments, the recovery of alloxydim deposited on carnauba wax and sandy loam soil surfaces was studied. The extraction procedure allows recovering between 99% and 102% of alloxydim from soil and carnauba surfaces, indicating that the herbicide is not irreversibly adsorbed in both matrices. Moreover, experimental runs in the dark at room temperature for 24 h did not show significant changes in the herbicide concentration, so alloxydim was found to be stable and reactions not initiated by sunlight can be discounted.

**Figure 1:**
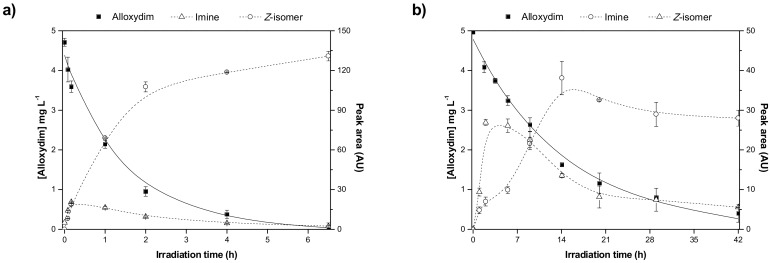
Kinetic evolution of the photodegradation products (open symbols and dotted lines) formed during the irradiation of alloxydim (filled symbols and solid line) on (**a**) carnauba wax films and (**b**) sandy loam soil.

Firstly, zero-order, first-order, and second-order kinetic models were used to check the kinetic order of alloxydim photolysis. Whereas data for alloxydim photolysis do not fit well to the linearized kinetic equations of zero-order and second-order, it was found that there was a higher linear relationship between the irradiation time and the logarithm of the remaining concentration of alloxydim. Thus, the photodegradation of alloxydim on carnauba wax and sandy loam soil surfaces was well described by pseudo-first-order kinetics, and good correlation coefficients were obtained (0.96) (Figure 1). The photolysis rate of alloxydim on soil surfaces (t_1/2_ = 9.7 h) is significantly slower than on carnauba wax film (t_1/2_ = 51 min). Thus, after 7 h of irradiation, the alloxydim deposited on carnauba was nearly completely degraded, whereas 60% of the initial concentration of the herbicide remained on soil surfaces. This result can be attributed to the soil particles that can attenuate about 90% of the incident radiation in the top 0.2 mm.

Compared with our previous studies carried out in aqueous media, we observed that the rate of photolysis of alloxydim on carnauba wax and soil surfaces was slower compared to water [17]. This decrease in the photodegradation rate could be due to physical partition in the wax layer or chemical adsorption by soil minerals. Other authors suggest the formation of herbicide aggregates would favor the intermolecular interactions, leading to the deactivation of excited states of the alloxydim rather than its photodegradation [25].

**Figure 2:**
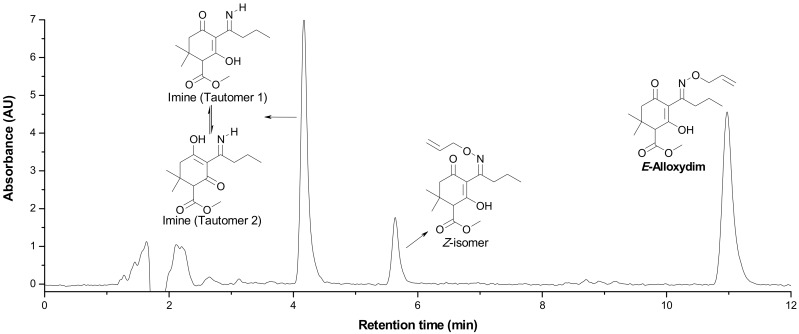
Representative HPLC-DAD chromatogram of alloxydim photodegraded on carnauba wax films and sandy loam soil under simulated sunlight (example on carnauba at irradiation time of 1 h).

As mentioned above, alloxydim was completely degraded on carnauba wax and soil surfaces, but the herbicide was not mineralized and different degradation photoproducts were detected in the course of the degradation process. Different samples from the photodegradation processes were analyzed by HPLC-MS to identify degradation products. The photodegradation of alloxydim in carnauba and soil showed the formation of two photoproducts with retention times of 4.2 and 5.8 min in the method employed. A typical chromatogram from an irradiated sample is shown in Figure 2.

These compounds showed *m*/*z* values of 323 and 268. By comparing their mass spectra with those of the previously reported work on degradation of alloxydim in aqueous media, we confirmed the structures of these byproducts as the *Z*-isomer and the imine byproduct. These byproducts were previously detected during the photodegradation of alloxydim in environmental waters [17,18], suggesting that the mechanism of photolysis could be the same due to the presence of moisture in the carnauba and soil surfaces. The imine byproduct identified as the major photoproduct was a mixture of two compounds (Figure 2), where each molecule presents a keto-enol tautomer. The equilibrium of the two tautomeric forms is shifted 63% towards tautomer 1 as a consequence of a hydrogen bridge between the oxygen of the ketone group and the hydrogen of the imine. The evolution of both degradation products in these matrices is shown in Figure 1. In both surfaces, the imine seems photostable and remains on carnauba and soil surfaces, whereas the *Z*-isomer was formed at the first stages of photolysis and subsequently disappeared slowly. These findings suggest that crops and soil not only could be exposed to the parent herbicide but also to its photoproducts, mainly the imine that could remain long enough to cause undesirable effects on nontarget organisms. Similarly, crops could be exposed to higher concentrations of the *Z*-alloxydim isomer since higher ratios of this compound were formed in carnauba wax films compared to soil (Figure 1).

In accordance with findings of other authors [11], the major degradation product is alloxydim imine. This byproduct is more soluble than alloxydim, which favors the presence of this byproduct in the water of the soil, and hence, its availability by root plants is higher. Furthermore, the imine molecule seems to be stable and could persist and accumulate in the environment, and thus, imine uptake by crop species could occur. This accumulation of imine would be greater in fields in which two or three crops are cultivated in one year.

Regarding the stability of the byproduct, its concentration remained constant during the whole bioassay experiment and the analysis did not show degradation or that its concentration was diminished.

### 2.2. Phytotoxicity Bioassay

The use of the bioassay technique, under optimum hydroponic conditions, promotes herbicide activity because the maximum herbicide bioavailability is facilitated to the plant. The most important parameter when measuring herbicidal effects is root length. The root is the first part of the plant affected by the herbicide, due to it being the site of uptake [26]. Although the results may be overestimations of risk, they may be useful as they offer a safety margin when extrapolating plant sensitivity to the field and the effect on crop production [27,28]. In a previous work of our research group, we observed that the main photoproduct of alloxydim shows a low phytotoxic effect on the germination of different seeds species [19].

In this sense, we have performed hydroponic bioassays of alloxydim and its imine byproduct on plant growth on two winter wheats, *Triticum turgidum* and *Triticum aestivum*, the grass weed *Bromus diandrus*, and two broadleaf crops, sugar beet and tomato. Wheat was selected as a species that is used as a successive and rotational crop with broadleaf crops. *Bromus diandrus* is a common grass weed found in Spanish fields, and tomato and sugar beet were selected to study the effect of a possible residue of alloxydim on those crops.

The results show that alloxydim has a strong post-emergent herbicidal activity on cereal crops and grass weed under controlled conditions, which could affect the agricultural yield of the rotational crop (Table 1).

After seven days of treatment, the most sensitive biological parameter for alloxydim was root length. In all species assayed, the root system of the control concentration presented normal growth (Table 1). Target plants (i.e., not sugar beet or tomato) dosed at higher concentrations than the control showed increased root deformity (main root twisted and lack of secondary roots), which could limit the growth and hence the production of these crops.

Wheat root growth was affected with alloxydim doses from 0.1 to 0.6 mg L^−1^. The root growth in wheat plants was reduced by 50% (EC_50_) at a dose of 0.14 mg L^−1^ for *Triticum turgidum* and 0.17 mg L^−1^ for *Triticum aestivum*. *Bromus diandrus* root growth was also increasingly affected from 0.1 to 0.6 mg L^−1^, and the EC_50_ value obtained was 0.23 mg L^−1^ (Table 1). In the same way, shoot length was increasingly reduced with alloxydim doses assayed from 0.1 to 0.6 mg L^−1^, causing a 50% reduction (EC_50_) in shoot length in *Triticum turgidum* at 0.33 mg L^−1^ and in *Triticum aestivum* at 0.43 mg L^−1^. *Bromus diandrus* shoot growth was also more affected as dosage increased, and the EC_50_ value was 0.46 mg L^−1^ (Table 1).

Based on the responses of root and shoot growth inhibition produced by alloxydim on wheat and grass weed, the same doses were chosen to study the imine byproduct effects. However, alloxydim imine did not produce any significant effect on root and shoot length of *Beta Bulgari* sans *L. esculentum* at the dosages assayed. Thus, we could not calculate the EC_50_ values for each parameter.

The herbicidal activity of alloxydim caused EC_50_ values on fresh biomass of wheat species and *Bromus diandrus* ranging from 0.36 to 0.74 mg L^−1^. Dry biomass effects on these species (EC_50_ values) were lower than fresh biomass and ranged from 0.23 to 0.76 mg L^−1^ (Table 1). However, alloxydim imine byproduct had no significant effect on either the dry or fresh biomass of wheat, *Bromus diandrus*, and sugar beet.

In both wheat varieties and the weed grass *Bromus diandrus,* we observed herbicidal activity of alloxydim as expected and the imine byproduct had no phytotoxic effect. Neither alloxydim nor imine produced effects on sugar beet growth.

However, unexpectedly, we observed that the imine demonstrated phytotoxicity to tomato during the early growth stages. An effect in all parameters measured caused significant reduction in shoot growth. Doses between 0.2 and 1.2 mg L^−1^ of imine increasingly affected the growth inhibition of tomato plants. The EC_50_ values calculated were 0.23 mg L^−1^ for root length, 0.61 mg L^−1^ for shoot length, 0.62 mg L^−1^ for fresh biomass, and 0.63 mg L^−1^ for dry biomass. These results indicate that the application of alloxydim can affect the agricultural yield of tomato indirectly as a consequence of the formation of its imine derivative when this crop is used not only as the main crop but also as a rotational or successive crop.

### 2.3. QSAR Estimations

One first remarkable issue at the beginning of this discussion comes from the fact that the QSAR models used presently do not take into account the possible contribution and variations among the respective isomers (i.e., *E*- and *Z*-alloxydim). Future QSAR models developed using well-defined theoretical descriptors obtained by quantum chemical methods could provide further and specific information in this regard [21,29].

Validation of the QSAR models is a complex process that should be considered specially in the pesticide risk assessment process in order to accurately predict the activity of pesticides. In this sense, Gramatica (2007) [30] and Tropsha (2010) [31] performed excellent reviews focused on the validation of the QSAR models. Recently, Villaverde et al. (2018) [32] also treated this concern in a work focused on nanopesticide risk assessment within the European legislative framework. Work such as those performed by Zhang et al. (2006) [33] and Melagraki et al. (2017) [34] provide interesting modeling approaches to predict the activity of chemicals, fulfilling the criteria for model validation. However, QSARs whose domain of applicability is not accurately demonstrated for a family of compounds can also be used if the mathematical algorithms (e.g., linear regression) are obtained from “training sets” of well-known data of compounds of similar molecular structure, providing preliminary insight into concerns about the cause of problems in the safe use of pesticides [20,35,36]. In this sense, a first approximation to the most significant in silico results of individual alloxydim and several photoproducts (i.e., imine tautomers 1 and 2) are presented in Table 2. Moreover, the amine byproduct from alloxydim (see structure within Table 2) was subjected to QSAR analysis for comparison (Table 2), taking into account that several previous works have shown its possible formation in other environmental conditions [37].

#### 2.3.1. Physicochemical Properties

The estimated melting point of alloxydim (Table 2) confirms its known liquid state. Estimated values of this property for alloxydim byproducts (i.e., imine tautomers 1 and 2 and amine) also seem to indicate the same aggregation state. The estimated boiling point for alloxydim is 430.5 °C. However, alloxydim is well known as a very thermolabile herbicide [11], so its decomposition starts before reaching this temperature. Little is known about the stability of the alloxydim byproducts shown in Table 2. Their boiling points seem to be between 380–405 °C. However, the earlier decomposition observed with the parent compound shows that caution should be taken when drawing conclusions based solely on this property.

The remaining physicochemical properties studied (Table 2) do not differ significantly among the different compounds analyzed. Although the viscosity and surface tension of the amine are the main changes, with values that are 49.8% and 11.4% smaller, respectively, the other compounds have absolute values in the same range.

#### 2.3.2. Environmental Fate and Transport Endpoints

The calculated VPs show that alloxydim and its photoproducts should exist mainly in the liquid phase (i.e., VP: 10^−5^–10^−7^), with the hypothetical presence of amine mostly in the gas phase. The alloxydim environmental partitioning is corroborated by VP values obtained experimentally and available in the open literature [11]. Moreover, ingestion and dermal contact should be considered the most probable exposure pathways of concern for alloxydim and both imines (VP < 10^−6^ Pa). This would not be the case for amine, to which workers and the general population can suffer exposure by inhalation. All compounds show Henry’s Law constants (HLCs) < 10^−2^ Pa m^3^ mol^−1^. Therefore, they are nonvolatile in water.

The long-range transport study of pesticides and their behavior in the atmosphere is a current concern during pesticide risk assessment because experimental studies are either too complex or not viable [29]. However, QSAR analysis can provide additional key information such as atmospheric oxidation half-lives with hydroxyl radicals (12 h/day; 1.5 × 10^6^ OH cm^−3^) and ozone (at 7 × 10^11^ mol cm^−3^). In the case of alloxydim and its byproducts, these data seem to exclude their long-range transport by air with rapid and moderate oxidation, i.e., <2 and 2–24 h, respectively (Table 2). These findings are in agreement with those oxidizing properties recognized at European levels for other compounds of the same family, such as clethodim and profoxydim [38,39].

Water solubilities show a surprising increase from the parent compound (i.e., slightly soluble) to degradation products (i.e., soluble for both tautomers of imine and very soluble for amine) as a consequence of their greater polar character. Also, the soil adsorption coefficient (K_oc_) shows a low value for alloxydim, decreasing even more and significantly for both imine tautomers with negligible sorption to soil. The amine should show a low sorption to soil, similar to alloxydim. Furthermore, alloxydim and its photoproduct are clearly classified as leachers by their GUS indexes (GUS index > 2.8). Therefore, the contamination risk of waters for human consumption is high with parent herbicide, and this risk increases with the photoproducts.

However, both bioconcentration factors (BCFs) and bioaccumulation factors (BAFs) are lower for all assayed byproducts in comparison with the parent compound alloxydim, and their values are on the same order of magnitude. Moreover, BCF and BAF are always <1000 L kg^−1^ on a wet-weight basis. Therefore, alloxydim and its photoproducts have low bioconcentration and bioaccumulation potentials in both higher and lower trophic levels. This finding agrees with results collected in the literature for other cyclohexanedione oxime herbicides [38,39].

All of these estimations should be taken into account for future risk assessment, in order to have a whole body of information in light of current scientific and technical knowledge. The aim is to determine if these byproducts are of concern as a consequence of their environmental fate and transport.

#### 2.3.3. Ecotoxicity

A higher growth inhibition effect on *Tetrahymena pyriformis* after 48 h (IGC_50_) was observed via alloxydim than via the photoproducts. The 50% lethal concentrations (LC_50_) for *Daphnia magna* and Fathead minnow were of a similar order of magnitude for alloxydim and its byproducts (Table 2). These results are not in agreement with those observed previously, where photodegraded solutions showed a higher toxicity to the bacteria *Vibrio fischeri* than the active substance alloxydim [17]. These discrepant results are related to different factors: (a) *Daphnia magna* and *Vibrio fischeri* are different target species; (b) photoproducts could show synergic effects; (c) the amine (with the minor LC_50_ value) could be in the medium, but the analytical techniques used for its detection cause displacement of the chemical equilibrium towards the imine byproduct; and (d) the QSAR results should be treated with caution since the mean absolute errors are higher than 15%. These ecotoxicological estimations, together with a more severe oral rat LD_50_, the prediction about positive developmental toxicity and the mutagenicity in the *Salmonella typhimurium* for alloxydim byproducts, indicate a clear necessity to perform a full experimental set of experimental ecotoxicological studies. In this sense, a more complete view of the problem is necessary to perform a future and conclusive risk assessment.

## 3. Materials and Methods 

### 3.1. Reagents

Alloxydim (methyl (*E*)-(*RS*)-3-[1-(allyloxyimino)butyl]-4-hydroxy-6,6-dimethyl-2-oxocyclohex-3-enecarboxylate) was purchased from Dr. Ehrenstorfer GmbH (Augsburg, Germany) (98% purity) (Figure 2). The main imine byproduct was obtained by irradiating alloxydim aqueous solutions for 110 h with a xenon arc lamp (λ > 290 nm; intensity = 750 W m^−2^), and the imine byproduct was extracted by solid phase extraction [17]. 

Both acetonitrile (HPLC Super Gradient grade) and methanol (HPLC grade) were acquired from Macron Fine Chemicals (Gliwice, Poland). Ultrapure water, used for LC mobile phase and aqueous solutions, was obtained from a Millipore system (Milli-Q-50 18 mO). Formic acid (p.a.) was acquired from Merck (Darmstadt, Germany).

Carnauba wax and sandy loam soil were purchased from Aldrich (Steinheim, Germany). The main physicochemical properties of soil were: pH 5.9 in 0.01 M CaCl_2_, a cation exchange capacity of 16.6 mval/100 g, 3.32% of organic carbon, 0.31% of Kjeldahl nitrogen, and 0.26% of total phosphorus.

### 3.2. Photodegradation Experiments

The study of the photolytic behavior of alloxydim was performed on carnauba wax films and sandy loam soil surfaces as surrogates of plant leaf cuticle and real soils, respectively. The photodegradation experiments were performed in a Suntest CPS+ sunlight simulator from Atlas (Linsengericht, Germany) equipped with a xenon arc lamp and a special UV glass filter restricting the transmission of wavelengths below 290 nm. Photochemical studies were performed at an irradiation intensity of 500 W m^−2^, and the temperature was kept at 20 ± 1 °C using an Atlas SunCool chiller unit. This device provides a spectral distribution close to natural sunlight and a constant irradiance that allows performance of experiments under reproducible irradiation conditions, avoiding variations caused by geographical situation, seasonal or climatic conditions.

Soil surfaces were prepared by placing 2 g of sandy loam soil directly in Petri dishes (Ø = 4 cm). To prepare carnauba wax films, 2 g of solid wax was placed in Petri dishes (Ø = 4 cm) and heated at 100 °C for 10 min.

To perform the photolysis of the herbicides in both surfaces, a methanolic solution of alloxydim (300 µL, 125 mg L^−1^) was pipetted onto Petri dishes. The solvent was evaporated at room temperature, leaving behind a thin layer of the herbicide. The dishes were exposed to simulated solar light for different time intervals. After irradiation, carnauba wax films were rinsed with methanol (2 × 2 mL) and ultrapure water (2 × 2 mL). Soil samples were transferred to centrifuge tubes and extracted with methanol (2 × 2 mL) and ultrapure water (2 × 2 mL). The soil extracts were then centrifuged for 5 min, and the supernatant was filtered through 0.45-µm PVDF filters. The extracts from carnauba and soil surfaces were analyzed by HPLC-DAD-MS to separate and follow the evolution of the photoproducts formed during photodegradation experiments. The analytical column used was a Waters Atlantis^®^ T3 3-μm column (4.6 mm × 150 mm) with an ODS precolumn (3 μm), and the mobile phase was a mixture of water acidified with 0.1% of formic acid (A) and acetonitrile (B). The following gradient was used: 50% B for 1.2 min, 50–60% B for 0.8 min, 60–70% B for 1.0 min, and held at 70% for 9 min. The flow rate was 1 mL min^−1^ and the injection volume was 20 μL.

The photodegradation kinetics were followed until less than 10% of the initial concentration of the herbicide remained. Photodegradation experiments were performed in duplicate, and the results presented correspond to the arithmetic mean of these two independent analyses. Moreover, experiments in the absence of radiation (dark control) were performed in parallel to the photodegradation experiments under each condition tested and with initial concentrations of the herbicide to evaluate whether processes other than photolysis occurred.

### 3.3. Growth Chamber Bioassay

The study was conducted with two winter wheat species [*Triticum turgidum* L. (cv. Nita) and *Triticum aestivum* L. (cv. Pavon)], one grass weed (*Bromus diandrus* L.), and two broadleaf crops [sugar beet, *Beta bulgaris* L. (cv. Dulzata) and tomato, *Lycopersicon esculentum* L. (cv. Marmande)].

To perform the bioassays, alloxydim and its imine byproduct were dissolved in Hewitt nutrient solution at pH 6.7, prepared with deionized water [40]. The doses assayed with alloxydim and imine were from 0.1 to 0.6 mg L^−1^ for wheat, and the doses assayed in *Bromus diandrus*, sugar beet, and tomato were from 0.2 to 1.2 mg L^−1^. Controls were included in each assay. The assays were conducted in a growth chamber with 16 h of light (100 μE m^−2^ s^−1^) at 24 ± 1 °C and 8 h of darkness at 15 ± 1 °C. All seeds were pre-germinated in Petri dishes, and seedlings were placed on plastic grids in pots. The number of seedlings depended on the species: five seedlings of wheat (both types) and *Bromus diandrus* per pot with 5 replications per dose, two seedlings of sugar beet and one seedling of tomato per pot with 10 replications per dose were used.

Six independent bioassays were tested for each species, three for alloxydim and three for the imine byproduct. A total of 30 bioassay trays were arranged in a randomized complete block in the chamber replicate three times under control conditions. According to preliminary assays, alloxydim and imine doses used in the main research project were 0, 0.1, 0.2, 0.3, 0.4, 0.5, and 0.6 mg L^−1^ for wheat species; and 0, 0.2, 0.4, 0.6, 0.8, 1.0, and 1.2 mg L^−1^ for *Bromus diandrus* L., sugar beet, and tomato. Plants were removed after 7 days, and the root lengths were determined. The plants were cut, shoots and roots were separated, and then shoot lengths were determined and fresh biomass measured; finally, plants were dried at 80 °C for 48 h, and dry biomass was obtained.

### 3.4. Statistical Analyses

One-way analyses of variance (ANOVA) were conducted to determine differences between treatments at the 0.05 significance level. Separation of means was assessed by post hoc comparison of means using the LSD test at *p* < 0.05 level of significance.

Nonlinear regression was used to fit the log-logistic model [41] to the root length, shoot length, fresh biomass, and dry biomass data over dose for each species. The mathematical expression relating the response Y to the herbicide dose X is:

LOG-LOGISTIC: Y = C + ((D − C)/(1 + exp (bln(X) − In (EC50 + 1))))(1)

where C is the lower asymptote, D is the upper asymptote, b is the slope of the curve around the EC_50_, and EC_50_ is the dose giving 50% of response.

All analyses were performed using Statgraphics Plus v.5.0 software (Copyright^©^ 1994–2000 Statistical Graphics Corp. STATPOINT Technologies, Inc., The Plains, VA, USA). 

### 3.5. QSAR Models

EPI Suite™ software was used to predict several physicochemical properties (melting and boiling points) and environmental fate and transport endpoints [vapor pressure (VP), Henry’s Law constant (HLC), water solubility, octanol–air partition coefficient (K_oa_), octanol–water partition coefficient (K_ow_), organic carbon partition/soil adsorption coefficient (K_oc_), fish bioconcentration and bioaccumulation factors (BCF and BAF, respectively), atmospheric oxidation potential with hydroxyl radicals and ozone, half-life of the aerobic biodegradation of chemicals in soil, and the GUS index (Groundwater Ubiquity Score; GUS = log (t_1/2 soil_)∙(4−log K_oc_)).

The Toxicity Estimation Software Tool (T.E.S.T.) v. 4.2.1 program was used to determine additional physicochemical properties (density, viscosity, flash point, surface tension, and thermal conductivity) and ecotoxicological endpoints (48-h *Tetrahymena pyriformis* IGC_50_, 48-h *Daphnia magna* LC_50_, 96-h Fathead minnow LC_50_, oral rat LD_50_, developmental toxicity, and Ames mutagenicity).

Calculations with both software were performed by following the models and methodologies used by Villaverde et al. (2018) [42].

## 4. Conclusions

In this work, we have studied the phototransformation of alloxydim at the surface of wax films to simulate the processes occurring on leaves and at soil surfaces. Alloxydim was applied at the field doses and the radiation intensity was similar to solar radiation in the region in order to mimic as much as possible the environmental conditions. The results showed that photodegradation is an important dissipation pathway of alloxydim in soil and plant leaf surrogates, with half-lives of less than one hour for the degradation on carnauba wax and 10 h on soil. Therefore, it is of great importance to know the photochemical behavior of alloxydim to avoid underestimating the consequences of its use. The reaction rates are higher than in aqueous media, which highlights the importance of performing these studies on the risk assessment of pesticides since it is not possible to extrapolate the photoreactivity on wax or soil from the water studies. The degradation products formed during alloxydim photodegradation were identified as the Z-isomer and an imine compound with a higher photostability than the parent compound. Further, bioassays on two target crops (i.e., sugar beet and tomato), rotational crops (two winter wheat species), and one grass weed showed that accumulation of imine byproduct could damage crops like tomato but not sugar beet. Furthermore, employing wheat as a rotational crop of broadleaf crops should not be affected by alloxydim residues. 

QSAR analysis for alloxydim photoproducts suggests that dermal contact and ingestion are the most probable exposure paths of concern for humans. The imine byproduct is considered as a leacher (GUS index > 2.8) due to the low sorption to soil and high solubility in water, highlighting the risk of contaminating ground water and the subsequent human consumption. In terms of ecotoxicity, QSAR predictions are inconclusive, indicating the necessity of a future experimental ecotoxicological study. 

It can be concluded that the findings in this study should encourage further attention to transformation products when evaluating pesticides’ risk.

In addition, QSAR models should be further developed to cover the gaps in the initial risk assessment regarding TPs, which in most cases are not available for experimental testing.

## Figures and Tables

**Table 1:** Regression equation (by Seefeldt equation) and EC_50_ values for alloxydim and the alloxydim imine tested in bioassay parameters that were the object of study ^†^.

		Alloxydim					Alloxydim Imine				
Parameter	Species	Upper Asymptote (C) (cm)	Lower Asymptote (D) (cm)	Slope (b) (cm mg^−1^ m^−1^ L^−1^)	EC_50_ (mg L^−1^)	R^2^ (%)	Upper Asymptote (C) (cm)	Lower Asymptote (D) (cm)	Slope (b) (cm mg^−1^ m^−1^ L^−1^)	EC_50_ (mg L^−1^)	R^2^ (%)
	*T. turgidum*	3.43	16.89	26.08	0.15	97	-	-	-	-	-
	*T. aestivum*	2.06	17.85	28.24	0.17	93	-	-	-	-	-
Root length	*B. diandrus*	3.57	7.94	17.16	0.24	83	-	-	-	-	-
	*B. vulgaris*	*n*/*a*					-	-	-	-	-
	*L. esculentum*	*n*/*a*					8.58	13.94	27.05	0.23	58
	*T. turgidum*	8.78	16.80	43.58	0.33	82	-	-	-	-	-
	*T. aestivum*	8.06	15.85	25.65	0.43	85	-	-	-	-	-
Shoot length	*B. diandrus*	7.32	13.38	9.40	0.46	82	-	-	-	-	-
	*B. vulgaris*	*n*/*a*					-	-	-	-	-
	*L. esculentum*	*n*/*a*					8.47	11.20	18.02	0.61	48
	*T. turgidum*	0.48	1.33	20.13	0.42	61	-	-	-	-	-
	*T. aestivum*	0.48	1.28	21.60	0.36	78	-	-	-	-	-
Fresh biomass	*B. diandrus*	0.24	0.54	4.91	0.74	84	-	-	-	-	-
	*B. vulgaris*	*n*/*a*					-	-	-	-	-
	*L. esculentum*	*n*/*a*					0.32	0.57	20.14	0.62	44
	*T. turgidum*	0.04	0.14	19.18	0.23	58	-	-	-	-	-
	*T. aestivum*	0.04	0.12	24.25	0.35	64	-	-	-	-	-
Dry biomass	*B. diandrus*	0.01	0.09	2.45	0.76	70	-	-	-	-	-
	*B. vulgaris*	*n*/*a*					-	-	-	-	-
	*L. esculentum*	*n*/*a*					0.03	0.05	18.46	0.63	29

^†^*n*/*a*: not applicable; -: not adjusted.

**Table 2:** Estimation of several physicochemical properties and environmental and ecotoxicological endpoints for alloxydim and its byproducts using EPI Suite^TM^ or T.E.S.T. models. The mean absolute errors are determined in both the entire training set and external test set of the T.E.S.T. models.

Endpoints	Models	*E*-, *Z*-Alloxydim 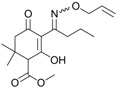	Imine (Tautomer 1) 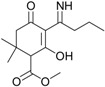	Imine (Tautomer 2) 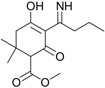	Amine 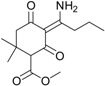
**Physicochemical properties**					
Melting point (°C)	EPISuite	162.4	152.1	152.1	139.1
Boiling point (°C)	EPISuite	430.5	405.8	405.8	381.3
Density (g cm^−^^3^)	T.E.S.T.	1.14 *	1.24 *	1.24 *	1.21 *
Viscosity at 25 °C (cP)	T.E.S.T.	9.54 *	9.88 *	8.38 *	4.96 **
Flash point (°C)	T.E.S.T.	204.4 *	204.0 *	202.1 *	187.5 *
Surface tension at 25 °C (dyn cm^−^^1^)	T.E.S.T.	35.4 *	35.5 *	36.9 *	32.7 *
Thermal conductivity at 25 °C (mW mK^−^^1^)	T.E.S.T.	142.0 *	143.7 *	143.5 *	136.8 *
**Environmental endpoints**					
VP at 25 °C (Pa)	EPISuite	1.2 × 10^−^^7^	8.5 × 10^−^^7^	8.5 × 10^−^^7^	2.2 × 10^−^^4^
HLC at 25 °C (Pa m^3^ mol^−^^1^)	EPISuite	2.7 × 10^−^^7^	3.3 × 10^−^^7^	3.3 × 10^−^^7^	1.6 × 10^−^^9^
t_1/2_/^•^OH (h)	EPISuite	0.984	1.23	1.23	1.63
t_1/2_/O_3_ (h)	EPISuite	11.77	24.18	24.18	24.18
t_1/2_/aerobic biodegradation (d)	EPISuite	51.6	40.2	40.2	55.3
Water solubility at 25 °C, (mg L^−^^1^)	EPISuite	37.69	1.04 × 10^4^	1.04 × 10^4^	5.18 × 10^4^
log K_OA_	EPISuite	12.74	10.18	10.18	12.85
log K_OW_	EPISuite	2.78	0.303	0.303	0.668
log K_OC_	EPISuite	2.18	0.810	0.810	2.11
BCF (L kg^−^^1^ wet-wt) ^†^	EPISuite	31.8	3.16	3.16	3.16
BAF (L kg^−^^1^ wet-wt) ^‡^	EPISuite	13.9	0.931	0.931	1.06
GUS index ^§^		3.11	5.12	5.12	3.30
**Ecotoxicological endpoints**					
*T. pyriformis* IGC_50_ (48 h) (mg L^−^^1^)	T.E.S.T.	9.19 **	32.8 *	44.9 *	16.6 *
*Daphnia magna* LC_50_ (48 h) (mg L^−^^1^)	T.E.S.T.	7.43 **	7.84 **	6.40 **	5.52 **
Fathead minnow LC_50_ (96 h) (mg L^−^^1^)	T.E.S.T.	3.05 **	4.55 **	4.02 **	4.40 **
Oral rat LD_50_ (mg kg^−^^1^)	T.E.S.T.	941.3 *	439.0 *	173.3 *	1562 *
Developmental toxicity	T.E.S.T.	Yes	Yes	Yes	Yes
Mutagenicity	T.E.S.T.	Positive	Positive	Positive	Positive

^†^ BCF: Fish bioconcentration factor; ^‡^ BAF: Fish bioaccumulation factor; ^§^ GUS: Groundwater ubiquity score. * Mean absolute error ≤ 15%, ** Mean absolute error > 15%.
